# Development and Validation of a Prognostic Classifier Based on Lipid Metabolism–Related Genes in Gastric Cancer

**DOI:** 10.3389/fmolb.2021.691143

**Published:** 2021-06-30

**Authors:** Xiao-Li Wei, Tian-Qi Luo, Jia-Ning Li, Zhi-Cheng Xue, Yun Wang, You Zhang, Ying-Bo Chen, Chuan Peng

**Affiliations:** ^1^ Department of Medical Oncology, Sun Yat-sen University Cancer Center, State Key Laboratory of Oncology in South China, Collaborative Innovation Center for Cancer Medicine, Guangzhou, China; ^2^ Department of Gastric Surgery, Sun Yat-sen University Cancer Center, State Key Laboratory of Oncology in South China, Collaborative Innovation Center for Cancer Medicine, Guangzhou, China; ^3^ Department of Clinical Research, Sun Yat-sen University Cancer Center, State Key Laboratory of Oncology in South China, Collaborative Innovation Center for Cancer Medicine, Guangzhou, China; ^4^ Department of Hematologic Oncology, Sun Yat-sen University Cancer Center, State Key Laboratory of Oncology in South China, Collaborative Innovation Center for Cancer Medicine, Guangzhou, China; ^5^ Zhongshan School of Medicine, Sun Yat-sen University Cancer Center, Guangzhou, China; ^6^ Department of Ultrasound, Sun Yat-sen University Cancer Center, State Key Laboratory of Oncology in South China, Collaborative Innovation Center for Cancer Medicine, Guangzhou, China

**Keywords:** gastric cancer, prognostic model, lipid metabolism, nomogram, gene expression omnibus dataset, gene panel

## Abstract

**Background:** Dysregulation of lipid metabolism plays important roles in the tumorigenesis and progression of gastric cancer (GC). The present study aimed to establish a prognostic model based on the lipid metabolism–related genes in GC patients.

**Materials and Methods:** Two GC datasets from the Gene Expression Atlas, GSE62254 (*n* = 300) and GSE26942 (*n* = 217), were used as training and validation cohorts to establish a risk predictive scoring model. The efficacy of this model was assessed by ROC analysis. The association of the risk predictive scores with patient characteristics and immune cell subtypes was evaluated. A nomogram was constructed based on the risk predictive score model and other prognostic factors.

**Results:** A risk predictive score model was established based on the expression of 19 lipid metabolism–related genes (LPL, IPMK, PLCB3, CDIPT, PIK3CA, DPM2, PIGZ, GPD2, GPX3, LTC4S, CYP1A2, GALC, SGMS1, SMPD2, SMPD3, FUT6, ST3GAL1, B4GALNT1, and ACADS). The time-dependent ROC analysis revealed that the risk predictive score model was stable and robust. Patients with high risk scores had significantly unfavorable overall survival compared with those with low risk scores in both the training and validation cohorts. A higher risk score was associated with more aggressive features, including a higher tumor grade, a more advanced TNM stage, and diffuse type of Lauren classification of GC. Moreover, distinct immune cell subtypes and signaling pathways were found between the high–risk and low–risk score groups. A nomogram containing patients’ age, tumor stage, adjuvant chemotherapy, and the risk predictive score could accurately predict the survival probability of patients at 1, 3, and 5 years.

**Conclusion**: A novel 19-gene risk predictive score model was developed based on the lipid metabolism–related genes, which could be a potential prognostic indicator and therapeutic target of GC.

## Introduction

Gastric cancer (GC) is one of the leading causes of cancer-related death worldwide, ranking the third in males and the fifth in females (Bray et al., 2018). Current treatments of GC, including surgery, chemotherapy, and targeted regimens, improve the survival of patients to some extent (Johnston and Beckman, 2019). New prognostic biomarkers remain needed to lower risk, stratify patients, and guide future research for potential new therapeutic targets.

Deregulation of lipid metabolism has a critical role in the promotion of tumorigenesis and tumor progression (Röhrig and Schulze, 2016; Yu et al., 2018; Yang et al., 2020; Esposito et al., 2019). It also participates in the regulation of T cell function, including T cell proliferation and differentiation (Lochner et al., 2015; Raud et al., 2018). Dysregulation of lipid metabolism contributes to various aspects of tumor growth (Lochner et al., 2015; Raud et al., 2018). Lipoproteins, high lipid droplets, and excessive cholesteryl ester storage are hallmarks of aggressiveness of cancers (Yue et al., 2014; Liu et al., 2017). Therefore, targeting deregulated lipid metabolism is a promising strategy for cancer treatment (Liu et al., 2017; Iannelli et al., 2018).

GC progression is closely associated with alterations of lipid metabolism. A low level of serum high-density lipoprotein predicted a high risk of GC development, a high rate of lymphatic and vascular invasion, an advanced nodal metastasis, and a poor prognosis in patients with GC (Guo et al., 2007; Tamura et al., 2012; Nam et al., 2019). Adipocytes and fatty acids fueled metastasis and conferred a poor prognosis of GC (Duan et al., 2016; Tan et al., 2018; Jiang et al., 2019). Various lipid metabolites and genes involved in lipid metabolism also shared some roles in GC tumorigenesis or progression (Abbassi-Ghadi et al., 2013; Tao et al., 2019; Huang et al., 2020; Zhang et al., 2020). For example, adipocytes promoted peritoneal metastasis of GC through reprogramming of fatty acid metabolism mediated by phosphatidylinositol transfer protein, cytoplasmic 1 (PITPNC1) (Tan et al., 2018). Enhanced fatty acid carnitinylation and oxidation mediated by carnitine palmitoyltransferase 1C (CPT1C) promoted proliferative ability of GC (Chen et al., 2020).

The mechanisms of deregulation of lipid metabolism in cancers are complicated, including alteration in pathways involved in *de novo* lipogenesis, lipid uptake, lipid storage, and lipolysis and generating enhanced synthesis, uptake, consumption, and storage of fatty acids (Liu et al., 2017). However, an overall view of the prognostic value of lipid metabolism–related genes in GC remained to be explored (Liu et al., 2017). Identification of genes associated with clinical outcomes is important for further research in this area. In the current study, lipid metabolism–related gene sets were extracted and analyzed for their prognostic value in patients with GC. A novel lipid metabolism–related gene panel was developed and validated for its capability of predicting patient outcomes.

## Materials and Methods

### Study Subjects

Two GEO (Gene Expression Omnibus, https://www.ncbi.nlm.nih.gov/geo/) datasets, GSE62254 and GSE26942, were used for analyses. Patients eligible for analyses were as follows: 1) histologically diagnosed with gastric adenocarcinoma, 2) having available mRNA expression data, and having 3) available complete clinical and survival information. There were 300 and 217 patients in the GSE62254 and GSE26942 datasets, respectively. 17 patients were excluded from the GSE26942 dataset due to not meeting the inclusion criteria, including 12 cases of normal tissue, 3 cases of gastric stromal tumor, and 2 cases without available survival information. Finally, the 300-patient cohort from the GSE62254 dataset was used as the training cohort for our risk predictive score model development, and the 200-patient cohort from the GSE26942 dataset was used as the validation cohort. The risk predictive score was also validated in another two public GC datasets, including the TCGA dataset (*n* = 350) and GSE84437 dataset (*n* = 432).

### Risk Predictive Model Development in the Training Cohort

The normalized gene expression data of the GSE62254 dataset were downloaded from GEO. Prognosis relevant genes from lipid metabolism–related gene sets were identified using the “survival” package. All the lipid metabolism–related genes were subjected to the univariate Cox regression model, and 63 genes were identified to be associated with overall survival (OS). The 63 genes were further subjected to the LASSO Cox regression model analysis using the glmnet package, and then 19 genes were selected for construction of the risk prognostic scoring system. Calculation of risk scores was performed using the generated coefficients and corresponding expression. According to the risk scores, patients were classified into low-risk and high-risk groups with a cut-off value (risk score = −3.793587), which best stratified patients with different OS.

### Risk Predictive Model Validation in the Validation Cohort

The same model and coefficients in the training cohort were applied in the validation cohort (GSE26942 dataset). The normalized gene expression data of GSE26942 were downloaded. The efficacy of risk score prognostic classification was evaluated by ROC analysis with the timeROC package. The survival analysis was conducted as mentioned above and was also validated with another two gene sets (TCGA GC and GSE84437).

### Risk Predictive Model Assessment

The timeROC package of R software was applied to perform the time-dependent receiver-operating characteristic curve (ROC) analysis. Survival analysis with Kaplan–Meier plots and the log-rank test, and the univariate and multivariate Cox hazard model were performed. GO and KEGG functional enrichment analyses were conducted through the R package clusterProfiler.

### Evaluating the Relevance of the Risk Predictive Model With Immune Cells

The expression matrices of GSE62254 and GSE26942 datasets were uploaded to CIBERSORT to determine the tumor-infiltrating immune cell fractions, which were calculated according to the LM22 signature with 1,000 permutations, as described previously (Newman et al., 2015). The genes and the immune cell markers are listed in Supplementary File 1. The immune cell subtype fractions were compared between the low–risk and high–risk score groups.

### Construction of a Nomogram

Independent prognostic factors were identified through univariate and multivariate Cox regression analysis. The independent prognostic factors were used to construct the prognostic nomogram, which assessed the OS probability at 1, 3, and 5 years with the “rms” package in R software.

### Statistical Analysis

Student’s *t*-test, the chi-squared test, and the Mann–Whitney U test were used to examine the differences between groups. Spearman analysis was adopted to assess the correlation between gene expression levels. All calculations were performed with R 3.5.3 software (http://www.R-project.org). A two-tailed *p* value < 0.05 was considered statistically significant.

## Results

### Construction of a Risk Prognostic Model Based on Lipid Metabolism–Related Genes

Human lipid metabolism–related pathways were downloaded from the KEGG (https://www.kegg.jp/) database. 13 lipid metabolism–related pathways were included for analysis (Supplementary Table S1). The characteristics of patients in the GSE62254 and GSE26942 datasets are listed in Supplementary Table S2. The study procedures are shown in Supplementary Figure S1.

The risk prognostic model was developed using the training dataset (GSE62254). The univariate Cox regression model was used to identify genes with prognostic relevance for overall survival (OS). As a result, 63 genes were found to have statistically significant relevance with OS, and their correlations with each other were validated (Supplementary Figure S2A). The LASSO algorithm was used to reduce overfitting and construct the model. The LASSO algorithm is known to have the following features: it is simple and visible, can reduce variance through reduction of coefficients, and can increase interpretability and decrease overfitting through eliminating irrelevant variables. The risk score was built using the LASSO Cox regression model (Supplementary Figures S2B,S2C). 19 genes were selected for the construction of the risk prognostic scoring system (Supplementary Table S3). The correlations between these genes are shown in Supplementary Figure 2D. The risk score was calculated as follows based on the 19 genes:

Risk score = (0.100 * LPL) + (−0.374 * IPMK) + (−0.122 * PLCB3) + (0.311 * CDIPT) + (0.146 * PIK3CA) + (−0.263 * DPM2) + (0.310 * PIGZ) + (−0.594 * GPD2) + (0.043 * GPX3) + (0.077 * LTC4S) + (−0.782 * CYP1A2) + (−0.102 * GALC) + (−0.189 * SGMS1) + (−0.061 * SMPD2) + (−0.184 * SMPD3) + (−0.081 * FUT6) + (0.130 * ST3GAL1) + (0.549 * B4GALNT1) + (−0.040 * ACADS).

### Validation of the Risk Prognostic Model and Its Efficiency

**FIGURE 1:**
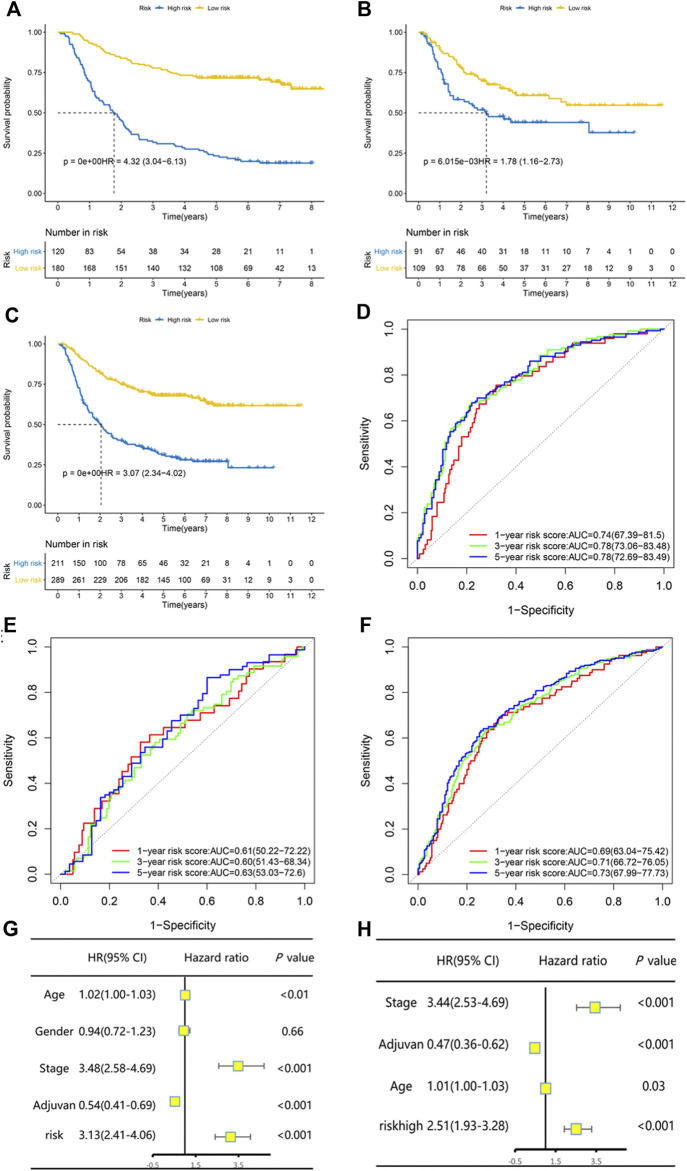
The risk predictive score model had high efficacy of prediction in the training set, the validation set, and the combination of both datasets. Kaplan–Meier curves of overall survival stratified by risk score (low/high) in the training set **(A)**, validation set **(B)**, and both datasets **(C)**. Time-dependent ROC analysis for the risk predictive model at 1, 3, and 5 years in the training set **(D)**, validation set **(E)**, and both datasets **(F)**. Univariate and multivariate Cox regression analysis for overall survival in both datasets **(G–H)**.

The GSE26942 dataset was adopted as the validation dataset. With a cut-off value of −3.793587, which stratified patients into two groups with the largest OS difference, patients were classified into low-risk and high-risk groups. The difference between the low-risk and high-risk groups in OS was statistically significant in the training dataset, the validation dataset, and both datasets combined. Kaplan–Meier curves are displayed in Figures 1A–C. Our constructed risk score also had significant prognostic relevance when validated with another two gene sets, TCGA GC and GSE84437 (Supplementary Figure S3). Subgroup analyses of Kaplan–Meier curves stratified by adjuvant chemotherapy (no/yes) and TNM stage (I + II/III + IV) in the combined dataset are displayed in Supplementary Figure S4. Time-dependent ROC analysis for the risk prognostic model at 1, 3, and 5 years is shown in Figures 1D–F. The area under the curve (AUC) was, respectively, 0.74 (95% CI: 0.67–0.82), 0.78 (95% CI: 0.73–0.83), and 0.78 (95% CI: 0.73–0.83) at 1, 3, and 5 years in the training dataset. The AUC was, respectively, 0.61 (95% CI: 0.50–0.72), 0.60 (95% CI: 0.51–0.68), and 0.63 (95% CI: 0.53–0.73) at 1, 3, and 5 years in the validation dataset and 0.69 (95% CI: 0.63–0.75), 0.71 (95% CI: 0.67–0.76), and 0.73 (95% CI: 0.68–0.77) at 1, 3, and 5 years in the combined dataset.

Univariate and multivariate Cox regression analysis in the combined dataset showed that the risk prognostic score model was an independent and significant prognostic factor for OS (Figures 1G,H). Patients with a high risk score had significantly worse OS (HR and 95% CI: 2.51 [1.93–3.28]) after being adjusted by other independent prognostic factors. The continuous patient risk score, survival state, and expression of the 19 genes of both datasets are shown in Supplementary Figure S5.

### Association of Risk Score With Clinical Characteristics and Immune Cells

**FIGURE 2:**
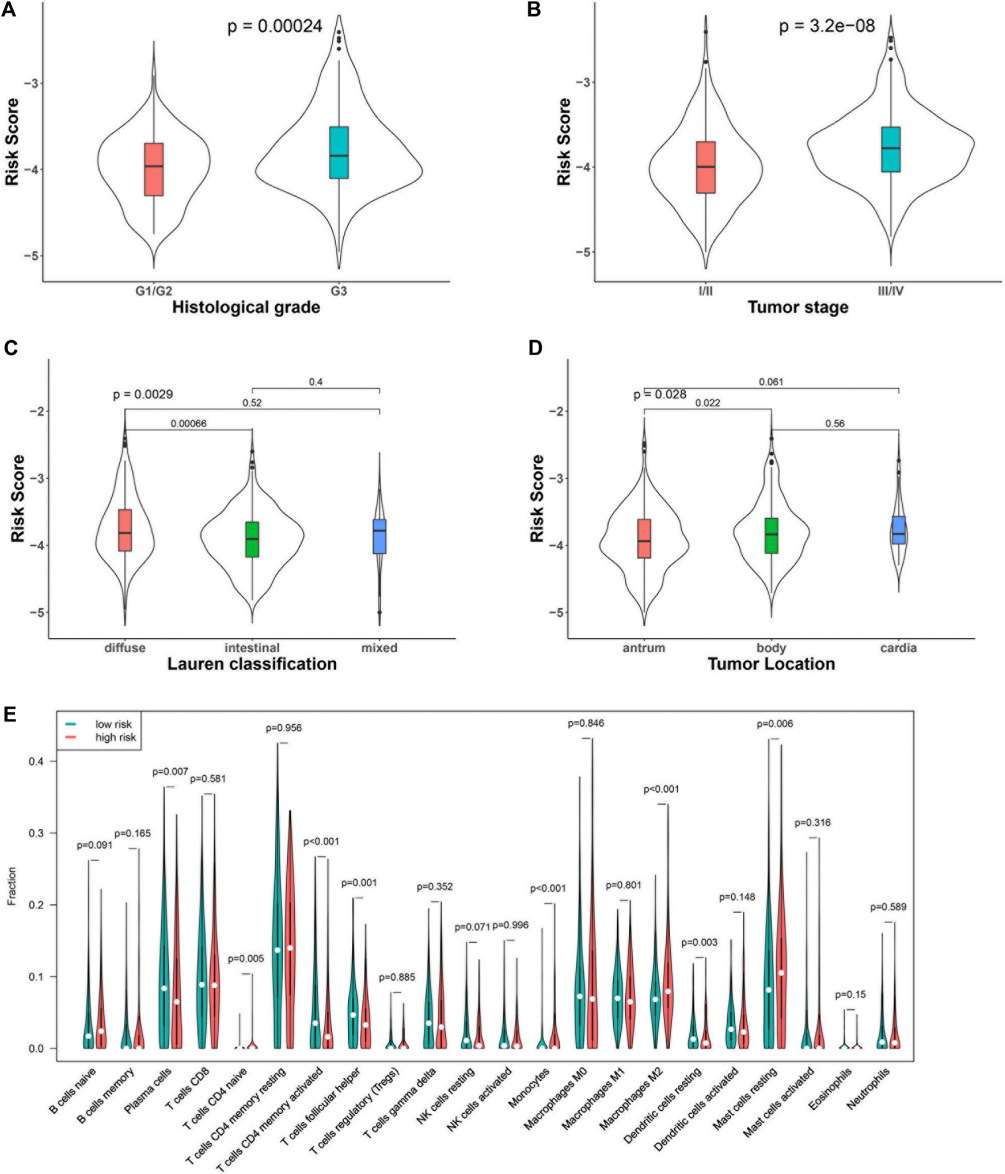
Association of the risk score with clinical characteristics and immune cells: tumor grade **(A)**, stage **(B)**, Lauren classification **(C)**, tumor location **(D)**, and immune cell subtypes **(E)**.

We compared the risk score between patients with different clinical characteristics. The results showed that the risk score between patients with different age (<60 or ≥60 years) was comparative (*p* = 0.11), so it was between males and females (*p* = 0.84). Tumors with more aggressive features generally had a higher risk score than those with less aggressive features. In particular, patients with a higher grade (G3) tumor had a higher risk score than those with a lower grade (G1/G2) tumor (*p* < 0.001, Figure 2A). Patients in stage Ⅲ/Ⅳ had a higher risk score than those in stage I/II (*p* < 0.001, Figure 2B). In terms of Lauren classification, diffused tumors had a higher risk score compared with the intestinal tumors (*p* < 0.001, Figure 2C). In addition, tumors located in the gastric body had a higher risk score than those located in the gastric antrum (*p* = 0.02, Figure 2D).

We analyzed the percentage of 22 immune cell subtypes in the tumors of both datasets and compared their level between patients with low and high risk scores (Figure 2E). The levels of some immune cells, including plasma cells (*p* = 0.007), activated CD4 memory cells (*p* < 0.001), follicular helper T cells (*p* = 0.001), and resting dendritic cells, were significantly lower in the high–risk score group (*p* = 0.003), while some immune cells, including naïve CD4 T cells (*p* = 0.005), monocytes (*p* < 0.001), M2 macrophages (*p* < 0.001), and resting mast cells, were higher in the high–risk score group (*p* = 0.006).

### Differentially Expressed Genes and Pathways Based on the Risk Prognostic Score

**FIGURE 3:**
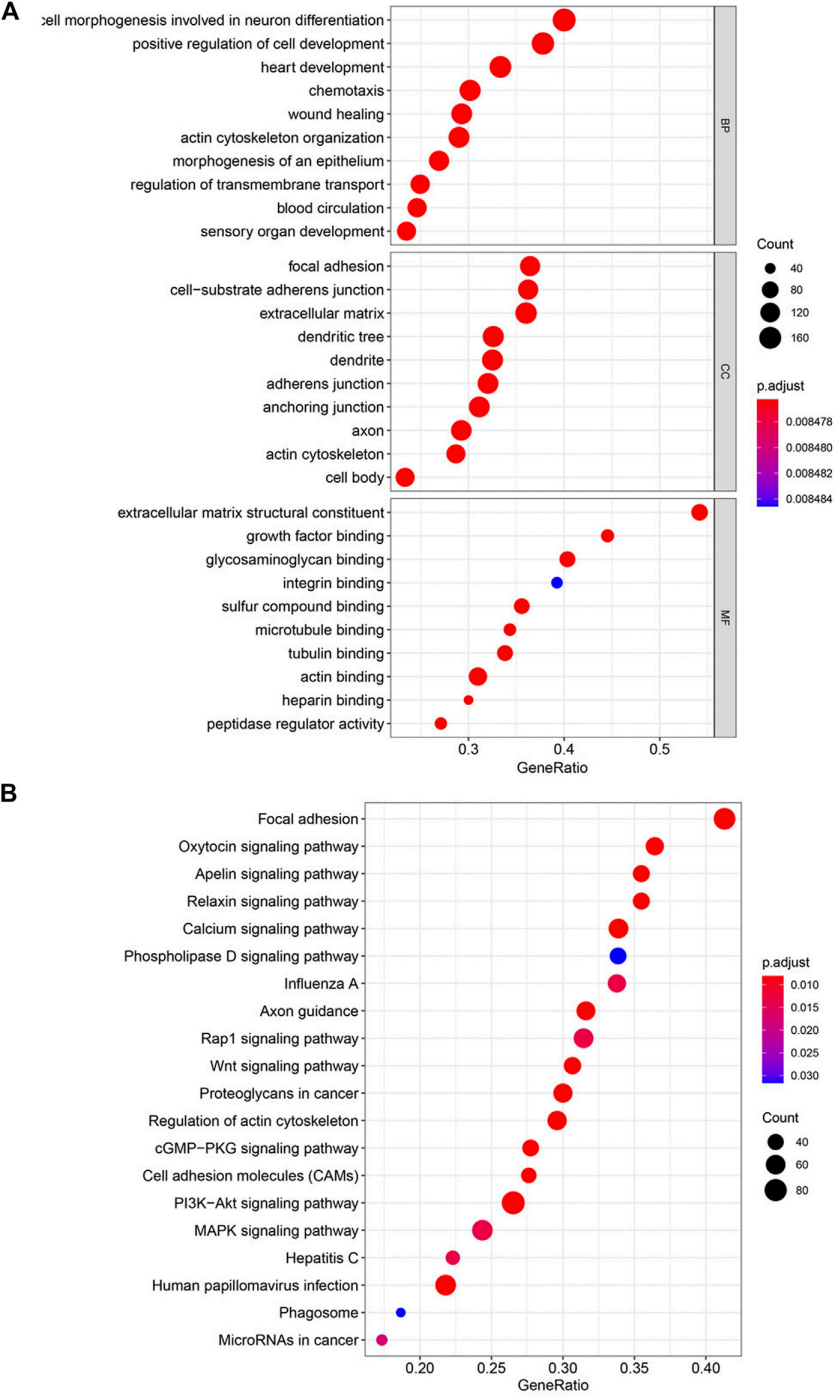
Enriched pathways by risk score with KEGG and GO functional enrichment analyses. **(A)** Top 10 GO terms in the biological process (BP), cellular component (CC), and molecular function (MF). **(B)** Top 20 enriched pathways in KEGG.

Functional analysis of differentially expressed genes was performed with KEGG and GO functional enrichment analyses. The top 10 GO genes were found to be associated with the biological process (BP), cellular component (CC), and molecular function (MF) (Figure 3A). These genes are associated with positive regulation of cell development, focal adhesion, cell-substrate adherens junction, extracellular matrix structural constituent, growth factor binding, etc. The top 20 enriched pathways are shown in Figure 3B, with the focal adhesion signaling pathway as the most significantly differently expressed pathway. Some other cancer-related pathways, including the Wnt signaling pathway, PI3K–Akt signaling pathway, and MAPK signaling pathway, were also significantly enriched in the high–risk score group.

### Construction of a Nomogram Model

**FIGURE 4:**
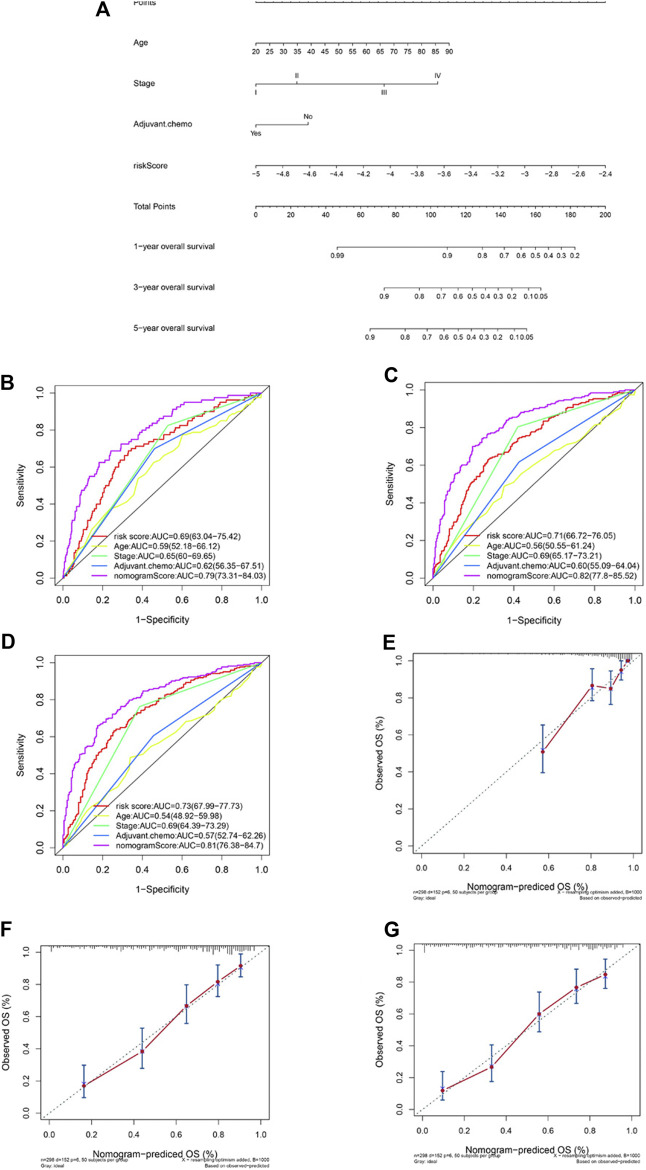
Construction of a nomogram based on the risk predictive score and other prognostic factors. Nomogram constructed based on the risk score and three other factors **(A)**. ROC analysis comparing the AUCs at survival of 1 year **(B)**, 3 years **(C),** and 5 years **(D)** among the nomogram, risk score model, age, stage, and adjuvant chemotherapy. Comparisons between the predicted overall survival of 1-year **(E)**, 3-year **(F)**, and 5-year **(G)** nomogram models and the ideal model.

Factors identified by univariate and multivariate Cox regression analysis as independent and significant prognostic factors were applied in the construction of a nomogram model (Figure 4A). As shown in Figures 1G,H, those factors included patients’ age, tumor stage, adjuvant chemotherapy, and the risk prognostic score. The predictive accuracy of the nomogram at 1 year (AUC = 0.79, Figure 4B), 3 years (AUC = 0.82, Figure 4C), and 5 years (AUC = 0.81, Figure 4D) was calculated and assessed by ROC analysis. The comparisons between the 1-, 3-, and 5-year nomogram models and the ideal model are shown in Figures 4E–G that displayed consistent indices and indicated relatively high accuracy of the nomogram models. The decision curve analysis (DCA) showed high predicting efficiency of the nomogram for the 3- and 5-year overall survival in the training dataset, the validation dataset, and the combination of both datasets (Supplementary Figures S6A–S6F).

## Discussion

GC has long been recognized as a recalcitrant cancer for its high incidence, high mortality, aggressive behavior, refractory traits, and poor prognosis (Van Cutsem et al., 2016). Identification of genetic factors that drive tumor progression and contribute to unfavorable outcomes is of key importance, for both improving patient care and developing potential therapeutics. As one of the most important basic metabolites, lipid has been demonstrated to be involved in the development and progression of malignancies in recent years, including GC (Huang et al., 2020). Although some studies have suggested important roles of lipid metabolites and lipid metabolism–related genes in GC (Huang et al., 2020), no reports have given an overall view of the prognostic value of lipid metabolism–related genes in GC.

In this study, we develop a novel prognostic scoring model based on the expression of lipid metabolism–related genes in gastric cancer. We used independent datasets from GEO to construct and validate the risk predictive scoring system containing 19 lipid metabolism–related genes. This scoring system was demonstrated to be efficient in predicting patient survival by ROC analysis. Patients had a significant and remarkable difference of OS between the high–risk and low–risk score groups. We further generated a nomogram integrating the risk predictive scoring system and three other prognostic factors (patients’ age, TNM stage, and adjuvant chemotherapy) that improved the efficiency of prognostic value of the nomogram and accurately predicted the 1-, 3-, and 5-year OS of GC patients in the GEO datasets.

The risk predictive score calculated with our scoring system was significantly associated with the aggressiveness of GC. Patients with a higher grade tumor and in an advanced stage were shown to have a higher risk score, suggesting that dysregulation of lipid metabolism not just was associated with cancer progression in GC but also served as a driving factor for the aggressiveness of GC. Some of the genes included in our risk scoring system had been found to be involved in cancers, such as lipoprotein lipase (LPL) (Zaidi et al., 2013), phosphate 3-kinase catalytic subunit alpha (PIK3CA) (Arafeh and Samuels, 2019), mitochondrial glycerol-3-phosphate dehydrogenase (GPD2) (Singh, 2014), leukotriene C4 synthase (LTC4S) (Halvorsen et al., 2014), galactosylceramidase (GALC) (Halvorsen et al., 2014), sphingomyelin phosphodiesterase 3 (SMPD3) (Singh et al., 2014), fucosyltransferase 6 (FUT6) (Singh et al., 2014), and β-galactoside α-2,3-sialyltransferase-1 (ST3Gal1) (Wu et al., 2018). Except for PIK3CA, which was found to be frequently altered in GC and was associated with unfavorable prognosis (Cancer Genome Atlas Research Network, 2014; Kim et al., 2017), although most of these genes were less studied, studies have suggested some roles of them in GC. LPL, which encodes lipoprotein lipase, a key enzyme in triglyceride metabolism, was reported to promote the progression of GC (Chang et al., 2017). Phospholipase C beta 3 (PLCB3), which encodes a member of the phosphoinositide phospholipase C beta enzyme family, was found to be one of the critical altered genes involved in aristolochic acid–induced gastric benign or malignant tumors (Wang et al., 2020). The polymorphism of cytochrome P450 family 1 subfamily A member 2 (CYP1A2) was repeatedly found to be associated with GC risk (Xue et al., 2014) and could modulate susceptibility to GC in patients with *Helicobacter pylori* infection (Ghoshal et al., 2014). Our study presented evidence for the prognostic value of these genes in GC, demonstrating their potential to be targets for anti-GC therapeutic research and development in the future.

Interestingly, the present study also revealed that patients with high and low risk scores had distinct features in tumor-infiltrating immune cells. Patients with high risk scores had significantly reduced number of plasma cells, activated CD4 memory cells, follicular helper T cells, and resting dendritic cells and increased number of naïve CD4 T cells, monocytes, M2 macrophages, and resting mast cells. Activated CD4 memory cells were associated with favorable outcomes in patients with cervical cancer (Ju et al., 2020) and favorable outcomes after radiotherapy in patients with multiple cancers (Ju et al., 2020; Wen et al., 2020). In gastric cancer, patients with low risk scores had increased number of activated CD4 memory cells and had superior prognosis (Zhao et al., 2020). Dendritic cells are specialized antigen-presenting cells which are key to the initiation of immune responses, including anti-tumor immune responses (Wculek et al., 2020). The increased number of dendritic cells induced by neoadjuvant chemotherapy was reported to be related to improved survival in GC (Hu et al., 2014). Naïve CD4 T cells were developed to form Treg cells in the tumor microenvironment and predicted poor prognosis in breast cancer (Su et al., 2017). M2 macrophages are a well-known tumor-promoting immunosuppressive cell type, and they have been proposed as a therapeutic target in GC (Gambardella et al., 2020). Immunotherapy has been established as a novel treatment in GC, but as monotherapy, PD-1 antibodies have limited benefit because the majority of patients do not respond (Xie et al., 2021). Novel combination options with immunotherapy are in great need in GC. Lipid metabolism not only impacts the proliferation and migration of tumor cells but also shapes the immuno-microenvironment by affecting the recruitment and function of tumor-infiltrating immune cells (He et al., 2021). In our study, patients with high risk scores had an immunosuppressive tumor microenvironment, indicating a possible role of treatments targeting lipid metabolism–related genes with immunotherapy in GC.

The major limitation of the present study was the lack of validation in larger patient cohorts from multicenter real-world clinical practice. Thus, the risk predictive score was still far from being able to be used in clinical practice. Another important limitation was that most of the genes used to construct this risk predictive score model were scarcely investigated in cancers. In addition, we did not perform the basic experiment to validate their roles and related mechanisms in GC cells. The biological mechanism was unclear and needed further experimental validation. However, as a prognostic risk score, our model was repeatedly validated and achieved consensus results, so the conclusions of our study are still convincing despite the lack of experimental validation of each gene’s role in GC.

## Conclusion

In the present study, a novel lipid metabolism–related gene-based risk predictive score model was constructed and validated in datasets of patients with GC. This risk predictive scoring system could efficiently predict patient outcomes and had significant correlation with immune cell subtypes. A nomogram containing the risk score was generated, and it improved the prognostic predictive value of the current TNM staging system. This study will be helpful in biomarker and therapeutics development for GC patients.

## Data Availability

The datasets presented in this study can be found in online repositories. The names of the repository/repositories and accession number(s) can be found in the article/Supplementary Material.

## Supplementary Material

Supplementary Figure 1 Flowchart of the study. Two GEO datasets, GSE62254 and GSE26942, were used as the training and validation datasets for the risk predictive score model construction. Further comparisons and establishment of a nomogram based on the risk scores were conducted.

Supplementary Figure 2 Construction of a risk predictive score model based on lipid metabolism–related genes. 63 prognostic relevant genes in lipid metabolism–related pathways were screened **(A)**. The risk predictive score system was constructed using the LASSO Cox regression model **(B,C)**. Correlation between the 19 selected genes **(D)**.

Supplementary Figure 3 Kaplan–Meier curves of overall survival stratified by risk score (low/high) in another two datasets: TCGA GC dataset **(A)** and GSE84437 dataset **(B)**.

Supplementary Figure 4 Subgroup analyses of Kaplan–Meier curves for overall survival stratified by adjuvant chemotherapy (no/yes) and TNM stage (I + II/III + IV) in the combined dataset. Adjuvant chemotherapy—no **(A)**, adjuvant chemotherapy—yes **(B)**, TNM stage—I + II **(C)**, and TNM stage—III + IV **(D)**.

Supplementary Figure 5 Expression of 19 genes **(A)**, continuous patient risk score **(B)**, and survival state **(C)** in both datasets.

Supplementary Figure 6 Decision curve analysis (DCA) for 3-year OS and 5-year OS. DCA for 3-year OS in the training dataset **(A)**, validation dataset **(B),** and both datasets **(C)**; DCA for 5-year OS in the training dataset **(D)**, validation dataset **(E),** and both datasets **(F)**.
