# Supplementary figures and images for: Development and Validation of a Prognostic Classifier Based on Lipid Metabolism–Related Genes in Gastric Cancer
Source: Front Mol Biosci. 2021 Jun 30;8:691143. doi: 10.3389/fmolb.2021.691143 (PMC8277939; doi:10.3389/fmolb.2021.691143)

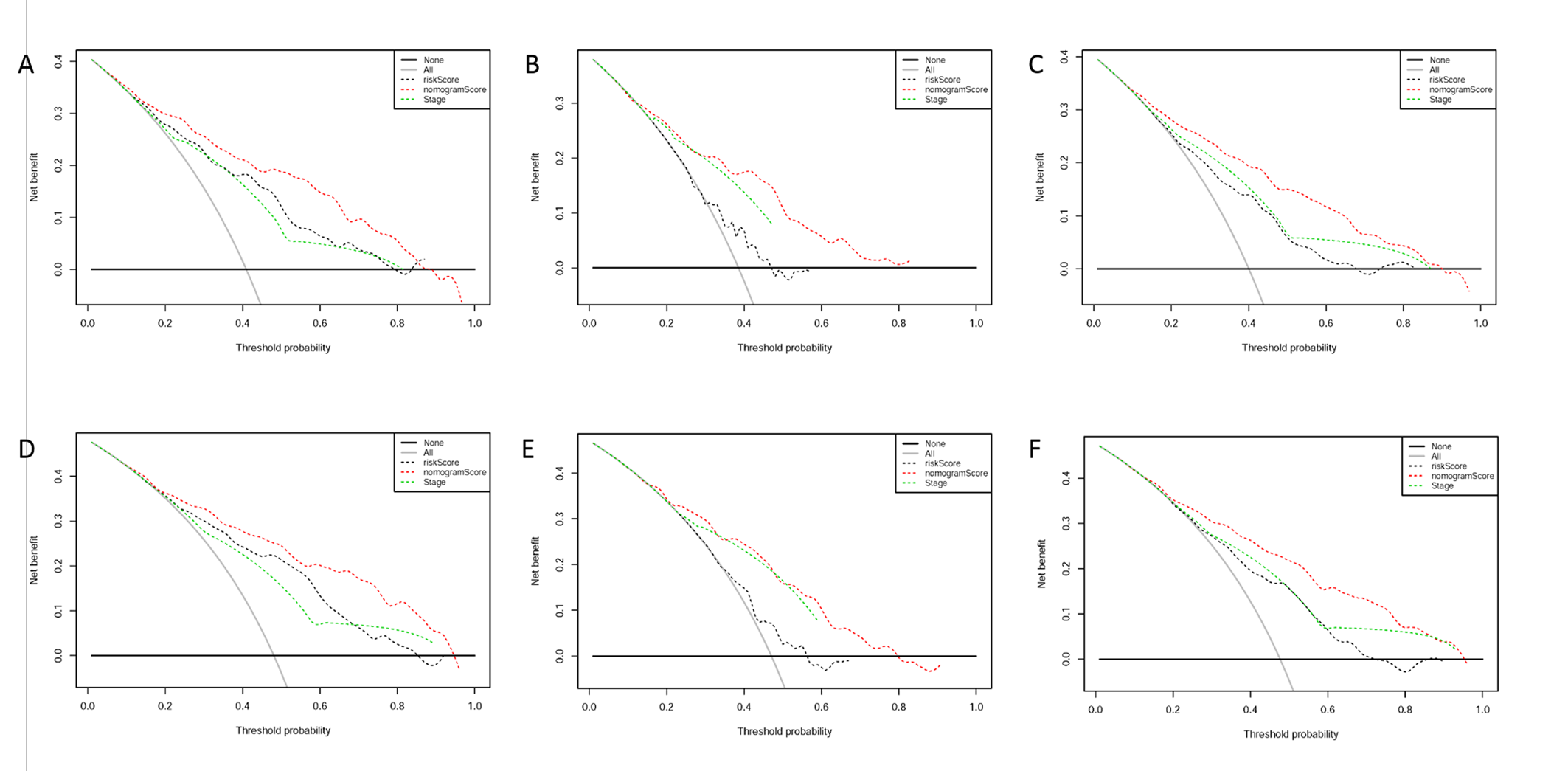

Supplement: Supplementary file 1 [file Image6.TIF]

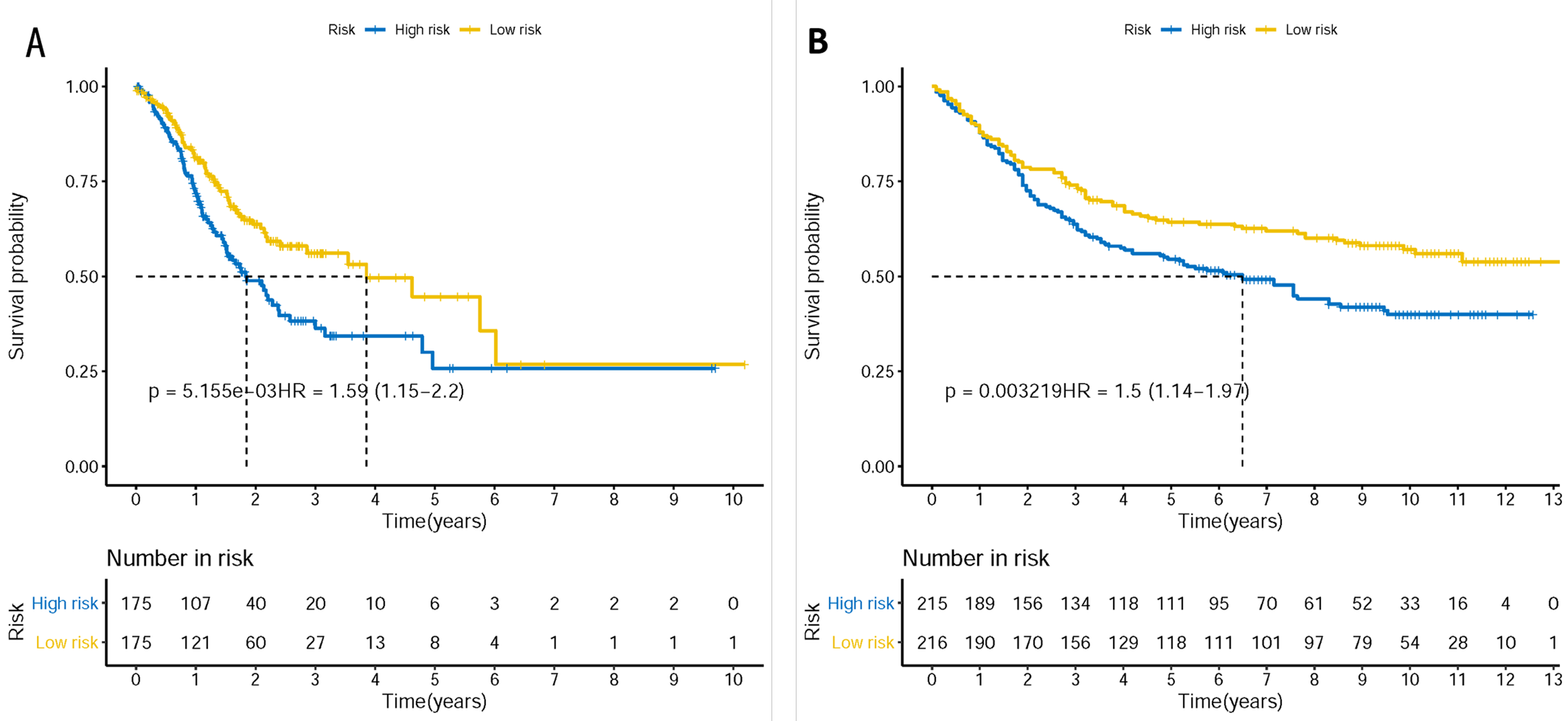

Supplement: Supplementary file 2 [file Image3.TIF]

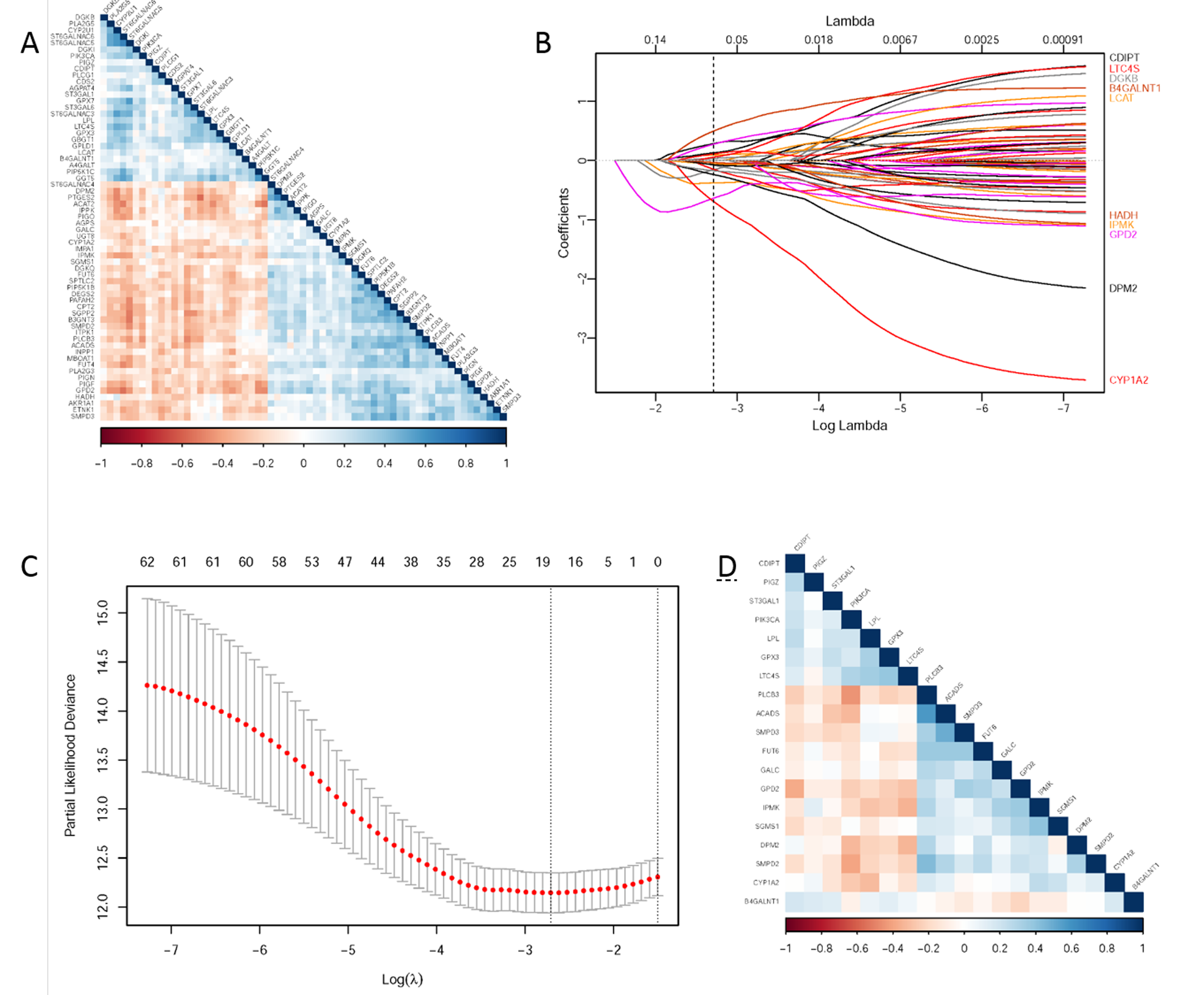

Supplement: Supplementary file 4 [file Image2.TIF]

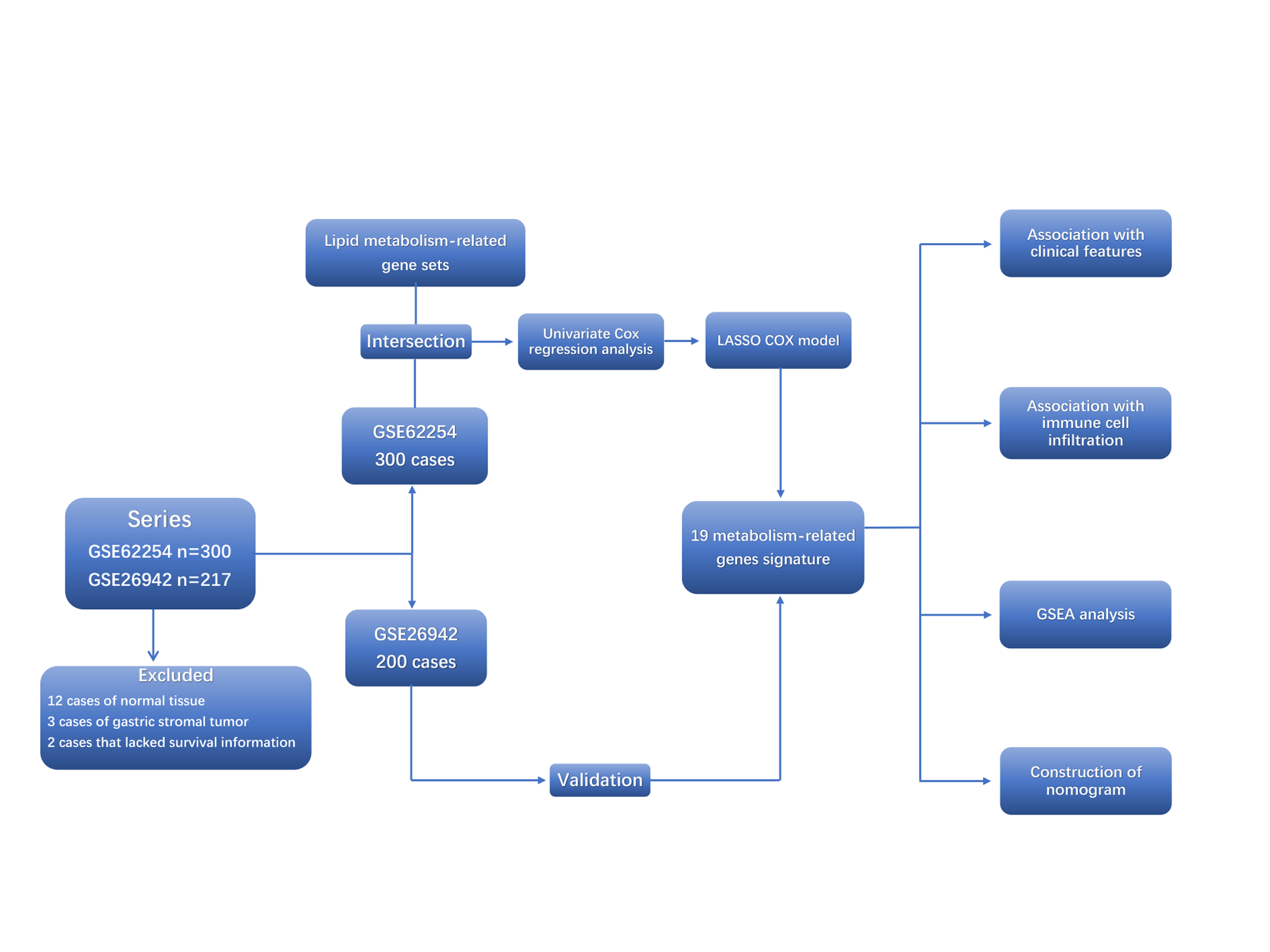

Supplement: Supplementary file 5 [file Image1.TIF]
