# Supplementary material for: Development and Validation of a Prognostic Classifier Based on Lipid Metabolism–Related Genes in Gastric Cancer
Source: Front Mol Biosci. 2021 Jun 30;8:691143. doi: 10.3389/fmolb.2021.691143 (PMC8277939; doi:10.3389/fmolb.2021.691143)
Supplement: Supplementary file 7 [file DataSheet1.docx]

Supplementary Table 1. Lipid metabolism-related pathways from KEGG.

|  | Lipid metabolism-related pathways |
| --- | --- |
| 1 | KEGG_GLYCEROLIPID_METABOLISM |
| 2 | KEGG_INOSITOL_PHOSPHATE_METABOLISM |
| 3 | KEGG_GLYCOSYLPHOSPHATIDYLINOSITOL_GPI_ANCHOR_BIOSYNTHESIS |
| 4 | KEGG_GLYCEROPHOSPHOLIPID_METABOLISM |
| 5 | KEGG_ETHER_LIPID_METABOLISM |
| 6 | KEGG_ARACHIDONIC_ACID_METABOLISM |
| 7 | KEGG_LINOLEIC_ACID_METABOLISM |
| 8 | KEGG_ALPHA_LINOLENIC_ACID_METABOLISM |
| 9 | KEGG_SPHINGOLIPID_METABOLISM |
| 10 | KEGG_GLYCOSPHINGOLIPID_BIOSYNTHESIS_LACTO_AND_NEOLACTO_SERIES |
| 11 | KEGG_GLYCOSPHINGOLIPID_BIOSYNTHESIS_GLOBO_SERIES |
| 12 | KEGG_GLYCOSPHINGOLIPID_BIOSYNTHESIS_GANGLIO_SERIES |
| 13 | KEGG_FATTY_ACID_METABOLISM |

Supplementary Table 2. Patient characteristics.

|  | GSE62254 (training cohort) | |  | GSE26942 (validation cohort) | |  |
| --- | --- | --- | --- | --- | --- | --- |
|  | high | low | p | high | low | p |
| n | 120 | 180 |  | 91 | 109 |  |
| riskScore (median [IQR]) | -3.53 [-3.66, -3.29] | -4.11 [-4.31, -3.96] | <0.001 | -3.53 [-3.68, -3.35] | -4.12 [-4.34, -3.93] | <0.001 |
| Age (median [IQR]) | 64.00 [53.00, 70.25] | 63.00 [56.00, 69.00] | 0.848 | 60.00 [50.00, 67.00] | 58.00 [50.00, 66.00] | 0.471 |
| Gender (%) |  |  | 0.432 |  |  | 0.386 |
| Female | 43 (35.8) | 58 (32.2) |  | 24 ( 26.4) | 35 ( 32.1) |  |
| Male | 77 (64.2) | 120 (66.7) |  | 66 ( 72.5) | 74 ( 67.9) |  |
| Unknown | 0 ( 0.0) | 2 ( 1.1) |  | 1 ( 1.1) | 0 ( 0.0) |  |
| Tumor stage |  |  | <0.001 |  |  | 0.005 |
| I | 3 ( 2.5) | 27 (15.0) |  | 12 ( 13.2) | 39 ( 35.8) |  |
| II | 29 (24.2) | 67 (37.2) |  | 18 ( 19.8) | 18 ( 16.5) |  |
| III | 73 (60.8) | 72 (40.0) |  | 52 ( 57.1) | 47 ( 43.1) |  |
| IV | 15 (12.5) | 12 ( 6.7) |  | 8 ( 8.8) | 5 ( 4.6) |  |
| Unknown | 0 ( 0.0) | 2 ( 1.1) |  | 1 ( 1.1) | 0 ( 0.0) |  |
| Lauren classification |  |  | 0.095 |  |  | 0.022 |
| diffuse | 60 (50.0) | 74 (41.1) |  | 25 ( 27.5) | 15 ( 13.8) |  |
| intestinal | 49 (40.8) | 97 (53.9) |  | 62 ( 68.1) | 79 ( 72.5) |  |
| mixed | 10 ( 8.3) | 7 ( 3.9) |  | 2 ( 2.2) | 5 ( 4.6) |  |
| Unknown | 1 ( 0.8) | 2 ( 1.1) |  | 2 ( 2.2) | 10 ( 9.2) |  |
| Adjuvant chemotherapy |  |  | 0.009 |  |  | 0.518 |
| Yes | 46 (38.3) | 98 (54.4) |  | 51 ( 56.0) | 55 ( 50.5) |  |
| No | 74 (61.7) | 82 (45.6) |  | 40 (44.0) | 54 (49.5) |  |
| Location (%) |  |  | 0.217 |  |  | 0.82 |
| antrum | 52 (43.3) | 98 (54.4) |  | 49 ( 53.8) | 60 ( 55.0) |  |
| body | 53 (44.2) | 64 (35.6) |  | 38 ( 41.8) | 46 ( 42.2) |  |
| cardia | 15 (12.5) | 17 ( 9.4) |  | 4 ( 4.4) | 3 ( 2.8) |  |
| Unknown | 0 ( 0.0) | 1 ( 0.6) |  | 0 ( 0.0) | 0 ( 0.0) |  |
| Overall survival |  |  | <0.001 |  |  | 0.065 |
| Alive | 23 (19.2) | 125 (69.4) |  | 44 (48.4) | 68 (62.4) |  |
| dead | 97 (80.8) | 55 (30.6) |  | 47 ( 51.6) | 41 ( 37.6) |  |
| Follow-up time | 1.77 [0.84, 4.71] | 5.60 [3.60, 6.93] | <0.001 | 2.16 [0.92, 4.32] | 3.86 [1.89, 6.99] | 0.001 |

Supplementary Table 3. 19 genes selected for the risk score system construction.

|  | Gene |
| --- | --- |
| 1 | LPL |
| 2 | IPMK |
| 3 | PLCB3 |
| 4 | CDIPT |
| 5 | PIK3CA |
| 6 | DPM2 |
| 7 | PIGZ |
| 8 | GPD2 |
| 9 | GPX3 |
| 10 | LTC4S |
| 11 | CYP1A2 |
| 12 | GALC |
| 13 | SGMS1 |
| 14 | SMPD2 |
| 15 | SMPD3 |
| 16 | FUT6 |
| 17 | ST3GAL1 |
| 18 | B4GALNT1 |
| 19 | ACADS |
